# 

**Published:** 1822-04

**Authors:** 


### MONTHLY CATALOGUE OF MEDICAL BOOKS.

The Medical Practitioner's Pocket Companion. 2s 6d.

An Essay on the Uterine Hæmorrhage which precedes the delivery of the full-grown Fetus. Illustrated with Cases. By Edward Rigby, M.D. 6th Ed. 7s.

Dr. Chisholm's Manual of the Climate and Diseases of Tropical Climates. 9s.

Mr. Lawrence's Lectures on Physiology, Zoology, &c. With Plates. 1l. 1s.

Observations on the Influence of Habits and Manners upon the Health and Organization of the Human Race. By Dr. Palin. 8vo. 10s. 6d.

Practical Rules for the Restoration and Preservation of Health. By the late George Cheyne, M.D. F.R.S. 4s. boards.

*Just imported, from America, by* J. SOUTER, 73, *St. Paul's Church-yard.*

| | £ | s. | d. |
|---|---|---|---|
| Chapman's Elements of Therapeutics and Materia Medica, Vol. I. Second Edition, enlarged. 8vo. bound | 0 | 16 | 0 |
| Silliman's Journal of Science and Arts. No. 1 to 4, each | 0 | 5 | 0 |
| ——— No. 5 to 9, (being to October 1821, inclusive,) each | 0 | 7 | 6 |
| Philadelphia Journal of Sciences. No. 1 to 5, (November 1821, inclusive,) each | 0 | 8 | 0 |
| New York Medical Repository, January to October 1821, (published every other month,) | 0 | 3 | 6 |
| The Emporium of Arts and Sciences. Vol. 1 to 3, New Series. 8vo. bds. | 2 | 14 | 0 |
| The Medical and Surgical Register, consisting of Cases in the New-York Hospital. Part 2. Vol. 1. 8vo. boards | 0 | 10 | 6 |
| Tracts on Medical Jurisprudence; with a Preface, Notes, and a Digest of the Law relating to Insanity and Nuisance. By Thos. Cooper, Esq. M.D. 8vo. bound | 0 | 18 | 0 |
| A General System of Toxicology, or a Treatise on Poisons; abridged and translated from the French of M. P. Orfila, M.D. by J. G. Nancrede, M.D. 8vo. bound | 0 | 18 | 0 |
| Rush's Medical Inquiries and Observations. 2 vols. 8vo. bound | 1 | 16 | 0 |
| A Memoir on Contagion, more especially as it respects the Yellow Fever. By N. Potter, M.D. 8vo. boards | 0 | 6 | 0 |
| History and Description of the Spotted Fever which prevailed at Gardener, Maine, in the Spring of 1814. By E. Hall, jun. M.D. M.M.S.S. 8vo. boards | 0 | 8 | 0 |
| A Discourse upon Vaccination. By Valentine Leaman, M.D. 8vo. bd. | 0 | 6 | 0 |
| A Compendium of Physiological and Systematic Botany; with Plates. By George Summer, M.D. 12mo. bound | 0 | 12 | 0 |
| A System of Anatomy for the use of Students of Medicine. By Caspar Wistar, M.D. 2 vols. 8vo. boards | 1 | 10 | 0 |
| Rafinesques Flora of the State of Louisiana. 12mo. sewed | 0 | 6 | 0 |
| American Medical Botany; being a Collection of the Medicinal Plants of the United States. By Jacob Bigelow, M.D. Vol. 1. two Parts, royal 4to. boards, each | 1 | 0 | 0 |

### NOTICES TO CORRESPONDENTS.

*We are compelled to postpone, for the present, the consideration of* Dr. EVANSON's *pamphlet.*

Mr. BUSH's *Communication came too late for insertion in the present Number. We cannot, however, forbear mentioning our decided aversion to all such controversies of a personal nature.*

A FRIEND *is informed that, on application to the proper quarter, we find that the term "dropsy," used in the enunciation of the question for the Fothergilian prize by the Medical Society of London, is intended to apply* comprehensively *to all kind of dropsies, in the same manner that nosologists employ the term "Hydrops."*

*We return thanks to the Registrar of the Medical Society of London, for the polite and ready manner in which he conveyed to us the information we required.*

Dr. DODS, *of Worcester, desires us to acquaint our readers that he will speedily publish a Second Part of his Physician's Guide.*

Mr. SHIPMAN, *of Guildford-street, is mistaken. The Reviewer of his book never* thought *that* Mr. SHIPMAN *"would prefer being abused to not being talked of at all,"* *as* Mr. SHIPMAN *plainly asserts in his letter to us. If he will have the goodness to read that review once more, he will find that the observation was made generally, and without reference to any particular individual.*

*We regret to have to announce the death of* Professor HALLÉ, *of Paris, one of the most distinguished practitioners of that metropolis.*

